# Commentary: Psychological well-being, mental distress, metabolic syndrome, and associated factors among people living in a refugee camp in Greece: a cross-sectional study

**DOI:** 10.3389/fpubh.2026.1764245

**Published:** 2026-02-11

**Authors:** Sandra Figueiredo, Alice Corradini

**Affiliations:** 1 University Research Center in Psychology (CUIP), Department of Psychology, Universidade Autónoma de Lisboa Luís de Camões (UAL), Lisbon, Portugal; 2 Department of Psychology, Università degli Studi di Perugia, Perugia, Italy

**Keywords:** psychologic adjustment, refugee, sleep disturbance, trauma, wellbeing

Despite cultural, social, and geographical differences, refugees share a common experience of forced displacement and exposure to severe adversity. Sleep disturbances constitute a core mechanism linking traumatic exposure to both physiological dysregulation and psychosocial maladjustment in forcibly displaced populations. Disrupted sleep has been consistently associated with alterations in stress regulation, metabolic functioning, and immune processes, as well as with heightened vulnerability to post-traumatic stress disorder (PTSD), depression, anxiety, and impaired daily functioning. In the context of refugees, sleep represents not merely a secondary symptom but a central pathway through which trauma and post-migration stressors affect overall wellbeing. While Knappe et al. (1) acknowledge insomnia as an associated factor in mental distress, their study engages with the extensive evidence linking trauma-related sleep disturbances to psychological and physical outcomes in refugee populations. Trauma is not confined to pre-migration but extends through dangerous migratory routes and post-migration conditions (2–4).

Although Knappe et al. (1) include insomnia among the associated factors examined. Their analytical approach reflects a broader trend in refugee mental health research that treats sleep disturbances as secondary or undifferentiated variables. In this study, sleep is introduced descriptively but not theoretically integrated, despite being repeatedly invoked in the interpretation of mental health deterioration. This conceptual ambiguity limits the explanatory value of insomnia as a predictor and weakens the internal coherence of the findings. This omission is evident in Knappe et al. (1). Since 2022, global crises such as the COVID-19 pandemic and the Ukraine–Russia conflict have further disrupted sleep. Pandemic-related isolation has altered circadian rhythms and daily routines (5, 6), and displacement has exacerbated these effects (7, 8). In Knappe et al. (1), sleep is treated as a secondary variable, particularly insomnia.

However, insomnia is repeatedly invoked in their discussion of mental health deterioration. Their approach adopts a simplified conceptualization of insomnia, treating it as uniform and ignoring its multidimensional and culturally variable nature in forcibly displaced groups. The study relies exclusively on self-reports and omits contextual factors such as noise, overcrowding, disrupted light cycles, and safety concerns, all of which are strong sleep determinants in refugee camps. No distinction is made between pre-, peri-, and post-migration sleep changes. Knappe et al. (1) do not distinguish between different phenotypes of sleep disturbance—such as difficulty initiating sleep, sleep maintenance problems, trauma-related nightmares, or nocturnal hyperarousal—despite evidence that these symptoms show distinct associations with PTSD, depression, and anxiety. As a result, clinically meaningful heterogeneity in trauma-related sleep disturbances is collapsed into a single insomnia indicator, limiting both its interpretability and translational relevance. However, Knappe et al. (1) do not situate their findings within this broader context of compounded global stressors, nor do they consider how such disruptions may have systematically influenced sleep-related self-reports, thereby introducing unexamined sources of bias.

Additionally, no mediational modeling is used to clarify how sleep interacts with PTSD, depression, anxiety, and stressors. Insomnia appears repeatedly as a statistically significant predictor of mental health problems, especially among women, which further highlights the need for a more nuanced treatment of this variable. The absence of considerations for cultural norms, co-sleeping, and age heterogeneity further limits interpretability. Although Knappe et al. (1) examine a wide range of associated factors, but their treatment of sleep-related variables lacks contextual and developmental sensitivity, particularly given the camp setting and the broad age range of participants. Insomnia should not be treated marginally, particularly in a sample spanning ages 16–59 living in a refugee camp in Greece. Age heterogeneity hinders conclusions about resilience and vulnerability.

Furthermore, cultural sleep practices and camp-related living conditions are not considered in the assessment of insomnia. Empirical studies conducted in refugee and forcibly displaced populations consistently document high rates of co-sleeping, shared sleeping spaces, and environmentally driven sleep disruption, including noise, overcrowding, and safety concerns. These factors have been shown to significantly influence sleep continuity, perceived sleep quality, and the expression of insomnia symptoms across age groups and cultural contexts. The absence of such empirically supported contextual variables further limits the ecological validity of the sleep-related conclusions drawn by Knappe et al. (1, 9).

The continued citation of this study (2023–2025) by others who also undervalue sleep raises methodological concerns. One-third of participants exceeded clinical thresholds for PTSD, depression, generalized anxiety disorder, and insomnia (1). Conclusions may be limited due to a lack of sleep variables. Physical activity was positively associated with sleep quality, demonstrating why sleep should be examined alongside behavioral factors. Insomnia emerges as a significant risk factor in post-migration adjustment (10), adding to existing burdens such as insecurity about asylum, family separation, language barriers, and discrimination (11).

Refugees exposed to traumatic events routinely show sleep disturbances (12–14). These long-standing findings should not be omitted. Research continuity requires revisiting and retesting well-established associations in current refugee contexts (15). Furthermore, metabolic risk is marginally addressed in Knappe et al., despite robust evidence linking sleep disturbances to adverse metabolic profiles (16, 17). Sleep disorders—insomnia, nightmares, fragmented sleep, early awakening, and non-restorative sleep—shape both psychological and physical functioning. Insomnia, defined by European guidelines as difficulty initiating or maintaining sleep or non-restorative rest (18), has severe daytime consequences (19) and complicates integration into host societies (20, 21). Post-migration stress is strongly associated with insomnia symptoms (22). However, Knappe et al. did not examine how specific post-migration stressors relate to insomnia (1).

Significant gaps remain in understanding sleep among forcibly displaced populations. We therefore recommend a re-analysis of Knappe et al.'s dataset with a focused examination of insomnia and its relationship to post-migration stressors, to support targeted interventions aimed at improving the wellbeing of refugees.
